# Variation in IgE binding potencies of seven Artemisia species depending on content of major allergens

**DOI:** 10.1186/s13601-020-00354-7

**Published:** 2020-11-18

**Authors:** Lan Zhao, Wanyi Fu, Biyuan Gao, Yi Liu, Shandong Wu, Zhi Chen, Xianqi Zhang, Huiying Wang, Yan Feng, Xueyan Wang, Hongtian Wang, Tianfei Lan, Meiling Liu, Xuefeng Wang, Yuemei Sun, Fangmei Luo, Gabriele Gadermaier, Fatima Ferreira, Serge A. Versteeg, Jaap H. Akkerdaas, Deyun Wang, Rudolf Valenta, Susanne Vrtala, Zhongshan Gao, Ronald van Ree

**Affiliations:** 1 Allergy Research Center, Zhejiang University, Hangzhou, 310058 China; 2 College of Agriculture and Biotechnology, Zhejiang University, Hangzhou, 310058 China; 3 State Key Laboratory of Respiratory Disease, The First Affiliated Hospital of Guangzhou Medical University, Guangzhou, 510120 China; 4 Hangzhou Aileji Biotech Ltd, Hangzhou, China; 5 School of Medicine, The First Affiliated Hospital, Zhejiang University, Hangzhou, 310013 China; 6 Department of Allergy, School of Medicine, the Second Affiliated Hospital, Zhejiang University, Hangzhou, 310013 China; 7 The First Affiliated Hospital, Shanxi Medical University, Taiyuan, Shanxi 030012 China; 8 Department of Allergy, Beijing Shijitan Hospital, Capital Medical University, Beijing, China; 9 Department of Allergy, The Third People’s Hospital of Datong, Datong, Shanxi 037008 China; 10 Department of Allergy, Yu Huang Ding Hospital, Yan Tai, Yantai, China; 11 Department of Otorhinolaryngology, Qvjing Chinese Traditional Medicine Hospital, Yunnan, China; 12 Department of Biosciences, University of Salzburg, Hellbrunnerstrasse 34, Salzburg, 5020 Austria; 13 Departments of Experimental Immunology and of Otorhinolaryngology, UMC, University Of Amsterdam, Meibergdreef 9, 1105 AZ , Amsterdam, The Netherlands; 14 Department of Otolaryngology, Yong Loo Lin School of Medicine, National University of Singapore, Singapore, Singapore; 15 Division of Immunopathology, Department of Pathophysiology and Allergy Research, Center for Pathophysiology, Infectiology and Immunology, Medical University of Vienna, Vienna, Austria; 16 Sechenov First Moscow State Medical University, Moscow, Russian Federation; 17 National Research Center - Institute of Immunology FMBA of Russia, Moscow, Russian Federation; 18 Karl Landsteiner University for Health Sciences, Krems, Austria

**Keywords:** Pollen allergen, Artemisia, Different species, IgE binding potency, Allergen quantification

## Abstract

**Background:**

*Artemisia* weed pollen allergy is important in the northern hemisphere. While over 350 species of this genus have been recorded, there has been no full investigation into whether different species may affect the allergen diagnosis and treatment. This study aimed to evaluate the variations in amino acid sequences and the content of major allergens, and how these affect specific IgE binding capacity in representative *Artemisia* species.

**Methods:**

Six representative *Artemisia* species from China and *Artemisia vulgaris* from Europe were used to determine allergen amino acid sequences by transcriptome, gene sequencing and mass spectrometry of the purified allergen component proteins. Sandwich ELISAs were developed and applied for Art v 1, Art v 2 and Art v 3 allergen quantification in different species. Aqueous pollen extracts and purified allergen components were used to assess IgE binding by ELISA and ImmunoCAP with mugwort allergic patient serum pools and individual sera from five areas in China.

**Results:**

The Art v 1 and Art v 2 homologous allergen sequences in the seven *Artemisia* species were highly conserved. Art v 3 type allergens in *A. annua* and *A. sieversiana* were more divergent compared to *A. argyi* and *A. vulgaris*. The allergen content of Art v 1 group in the seven extracts ranged from 3.4% to 7.1%, that of Art v 2 from 1.0% to 3.6%, and Art v 3 from 0.3% to 10.5%. The highest IgE binding potency for most Chinese Artemisia allergy patients was with *A. annua* pollen extract, followed by *A. vulgaris* and *A. argyi*, with *A. sieversiana* significantly lower. Natural Art v 1-3 isoallergens from different species have almost equivalent IgE binding capacity in *Artemisia* allergic patients from China.

**Conclusion and clinical relevance:**

There was high sequence similarity but different content of the three group allergens from different *Artemisia* species. Choice of *Artemisia annua* and *A. argyi* pollen source for diagnosis and immunotherapy is recommended in China.

## Background

*Artemisia* species are wind-pollinated weeds, widely distributed in the northern hemisphere with a few species in the southern hemisphere [1]. Pollens of *Artemisia* have been recognized as a major cause of late summer and autumn seasonal allergic respiratory disease, especially along the Asia-Europe silk-road and in north-western United States [1–6]. Between 350 and 500 *Artemisia* species have been recorded in the plant kingdom [1, 7] worldwide, 187 in China [8]. Phylogeny of the *Artemisia* genus, updated by molecular marker analysis [7, 9], has reached a consensus of six sections: Artemisia, Abrotanum, Dracunculus, Absinthium, Seriphidium and Tridentata. Most *Artemisia* species are in the first four sections and are distributed in temperate climate regions, where the majority of mugwort pollen allergic patients live. The few species belonging to Seriphidium and Tridentata are distributed in semi-desertic to steppic environments [10]. Some *Artemisia* species are dominant in natural plantations, contributing to the geographic difference of the pollen allergy [5]. *Artemisia vulgaris* is the best studied species, mainly distributed in northwestern and central Europe. Five major species have been listed in China (*A. annua, argyi, sieversiana, capillaris, lavandulifolia*) in a national pollen survey [11], and there is preliminary clinical and immunological evidence of the potential IgE binding potency of the first three species [12, 13]. A few species, such as *A. annua*, have invaded Europe and America, becoming potentially severe allergenic sources [14]. *Artemisia* pollen allergy is directly related to the distribution of *Artemisia* spp., density, climate [6] and risk factors [15]. Currently, commercial mugwort pollen allergen extract CAPs are from *A. absinthium* (w5) and *A. vulgaris* (w6), the latter being the most commonly used in diagnosis.

Molecular characterization of *Artemisia vulgaris* and *Artemisia annua* has revealed seven allergens, with the clinical data and reference DNA and protein sequences published [16, 17]. Art v 1 and Art v 3 have been shown to be major allergens worldwide, and a newly identified group, Art an 7 also seems to be important, although its IgE values are usually much lower [3, 18–20]. By sequence cloning of a single species of *Artemisia vulgaris* pollen, seven Art v 1 isoforms have been identified, with only slight variation in the C-terminal and very similar IgE reactivity [21]. Five Art v 3 isoforms have also been identified, one a partial sequence by N-terminal sequencing [22] and the other four by gene cloning [23]. Diversity of group 7 allergen sequences of seven *Artemisia* species has recently been reported, where two isoforms for each species have been found with over 95% identical sequence [17].

The current commercial mugwort pollen extract used for skin prick and immunotherapy in China is mainly from *A. sieversiana*, even though *A. annua* was recognized as an important allergen source in the 1980 s [5], and a recent report states that a mixture of pollens from three species (*argyi, annua, sieversiana*) would be better for immunotherapy (Bai et al. China Patent, CN102512673B). With a serum pool from the USA, high levels of cross-reactivity has been found with ELISA inhibition in nine *Artemisia* species, with two local sage species being the strongest inhibitors [1]. Very recently, using immunoblots, similar IgE binding patterns of seven *Artemisia* species have been found, with some degree of difference in three major allergen bands [17].

Cross-reactivity has been found in different *Artemisia* species [1], but whether different species in China have an impact on the allergen diagnosis and treatment has not been fully investigated. This study aims to provide a comprehensive analysis of sequence variation of different isoforms and variants, content of allergens Art v 1, Art v 2 and Art v 3, and their impact on IgE binding of six representative *Artemisia* species in China.

## Materials and methods

**Fig. 1:**
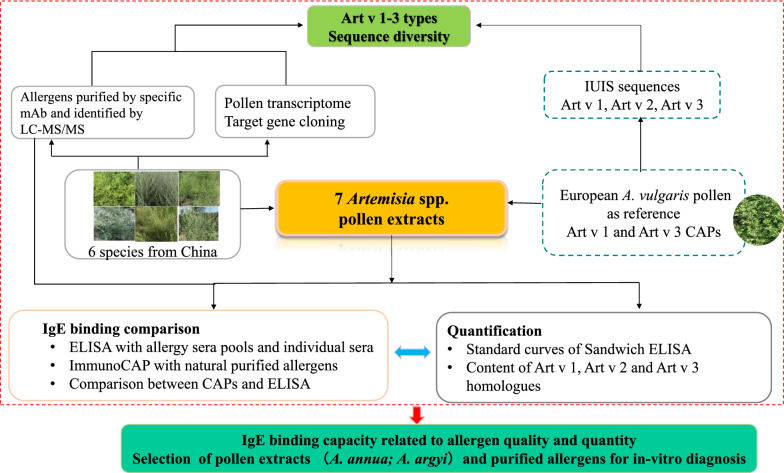
Overview of research design and outcomes

A graphic research design is presented in Fig. 1, with detailed information given in the following sections.

### *Artemisia s*pecies and protein extract

We used pollens of seven *Artemisia* species: five dominant in Northern China (*A. annua*, *A. argyi*, *A. capillaris*, *A. sieversiana*, *A. gmelinii*) collected from Shanxi Province; *A. lavandulifolia* from Zhejiang Province in South China collected over three years (Additional file 1: Figure S1), and *Artemisia vulgaris* pollen purchased from Allergon in Sweden. Species authenticity was verified by cloning and sequencing the ITS2 on genomic DNA from pollen according to a reported method [24]. Aqueous protein extracts of pollen were prepared by resuspending 0.2 g pollen grains in 3.5 ml PBS or 2 g in 35 ml PBS buffer (0.14 M NaCl, 2.7 mM KCl, 7.8 mM Na_2_HPO_4_, 1.5 mM KH_2_PO_4_), and shaking for 12 h at 4 °C. Extracts were centrifuged at 10,000 *g* for 10 min at 4 °C, filtered through 0.22 µm filters (Millipore), and the concentration of protein in extracts was determined using the BCA protein assay kit (Takara Bio, Japan). Three independent extracts from different pollen samples of each species from China, and one *A. vulgaris* sample were prepared and used in whole protein and individual allergen quantification. The rArt v 1.0101 and rArt v 3.0201 standards were from previous studies [21, 23].

### RNA extraction and Transcriptome

Total RNA was extracted from pollens of six *Artemisia* spp., collected from China, using the RNAprep pure kit (Tiangen, China) [17], and sequenced by BGI-Shenzhen and Hangzhou One Gene Ltd using Illumina HiSeq™ 2000 (San Diego, CA, USA). De novo transcriptome assembly was using the Trinity software package with a minimum K-mer of 3 and a minimum contig size of 100 bp. After extraction of allergenic protein sequences, blastx was used for alignment (e-value, 1e^−5^) between Unigenes and protein databases from the *Artemisia annua* genome [25].

### Cloning of Art v 1, Art v 2 and Art v 3 homologues

Pollen cDNA was prepared with the PrimeScript™ RT reagent kit with gDNA Eraser (Takara Bio, Japan) using mRNA fragments as templates. The full length of Art v 1, Art v 2 and Art v 3 homologues were obtained by PCR using primers based on Art v 1.0101 (AF493943) sequences: Art v 1-For, 5´-AATGGCAAAGTGTTCATATGTT-3´, Art v 1 - Rev, 5´-TTAGTGAGTGGACGGAGGAG-3´; Art v 2 sequence (AM279693) Art v 2-For, CCTCATACAGAAACATGGGAC, Art v 2-Rev, TTAGTAAGGTTTCTGACCAACAAC; and Art v 3.02 (EU564845, EU454846 original sequences provided by Gabriele Gadermaier) Art v 3-For, 5´-ATGGCAATGAAAATGATGAA-3´ and Art v 3- Rev, 5´-CTAGCATAAAGYAYTTCAC-3´for first round cloning. According to newly assembled transcript sequences for Art v 3 group, additional cloning for *A. capillaris* with AC-LTP-For: ATGGCAATRAAAATGATGAAGG, AC-LTP-Rev: TTCCATGTATTCCAGCATAAA; and *A. sieversiana* with AS-LTP-For: ATGGCAATGAAAATGATGAAG, AS-LTP-Rev: TCATTTCACCTTGTTGCAATC. PCR amplification with both primer pairs was using Phusion High-Fidelity DNA Polymerase (New England Biolabs, USA). At least eight clones were selected for sequencing. Nucleotide sequences and deduced amino acid sequences from different pollens have been deposited in GenBank. Isoallergens and variants were named following the nomenclature and the updated official list of the WHO/IUIS Allergen Nomenclature Sub-committee [26] and have been approved.

### Natural allergen purification and protein identity

Monoclonal antibodies (mAb) used in this study were from previous research [27]. A7-G4-E6 specific to Art v 1, C9-C1 to Art v 2, and A2-B8 to Art v 3, were used to purify three groups of allergens from six selected *Artemisia* spp. from China as described previously [27]. LC–MS/MS (Thermo Scientific Q Exactive) was used for identity-matching of the purified proteins to deduced allergens from each species. The purity of natural allergens was estimated by SDS-PAGE.

### Quantification of three components by ELISA

*A. argyi* extract was used to immunize two New Zealand rabbits to produce polyclonal antibodies (pAb), injecting with 500 μg protein in incomplete Freund’s adjuvant, followed by three subcutaneous boosters of 250 μg protein at intervals of 7-14 days. The quality was checked using both Western blot and ELISA. The antibodies were produced by Hua An Biotech Ltd., Hangzhou, China. An mAb (A7-G4-E6) and rabbit pAbs were used for quantification of Art v 1 homologous proteins. A mAb (C9-C1) and rabbit pAbs were used for quantification of Art v 2 homologous proteins. A selected mAb pair (A2-B8 and biotinylated A9-G10) ELISA assay was used to quantify Art v 3 homologous proteins with different recombinant or natural allergen standards. ELISA plates (Corning, USA) were coated with 0.3 μg capture antibodies (A7-G4-E6, C9-C1, A2-B8) at 4 °C overnight, after blocking with 100 μL 5% skimmed milk at 37 °C for 1 h, 100 μL serially diluted allergen standards and pollen extracts were added and incubated at 37°C for 1 h. After washing, the wells were incubated with 0.3 μg biotinylated detection antibodies at 37 °C for 1 h followed by incubation with 100 μL HRP-conjugated Streptavidin (1:5000 dilution) at 37 °C for 1 h. Finally, 100 μL TMB (3, 3′, 5, 5′-tetramethylbenzidine) was added as colorimetric substrate, and after incubation in the dark for 10 min, the reaction was stopped by adding 50 μL 2 M HCl. The optical density was measured at 450/620 nm (MultSkan FC, Thermo Fisher, USA). For each species, the allergen content was measured using three independent extracts with duplicate wells.

### Patients

A total of 150 patients (Additional file 1: Table S1**)** allergic to mugwort were recruited from Datong-Shanxi (111); Taiyuan-Shanxi (11); Beijing (10); Yantai-Shandong (10), and Qvjing-Yunnan (8) in China based on a convincing case history and positive IgE reactivity to mugwort extracts determined by ImmunoCAP (Thermo Fisher Scientific, Uppsala, Sweden). Eighty-two of these patients have been reported previously [17, 20, 28]. Specific IgE to the major mugwort allergen components, Art v 1 and Art v 3 was determined by ImmunoCAP. Individual sera and serum pools from the five areas were used to assess IgE binding capacity. The sera of five non-atopic individuals were pooled and used as a negative control. Written consent was obtained from all participants (or their representatives) and the study was approved by the local ethics committee.

### ELISA binding and inhibition analyses

Pollen extracts from the seven species were used to analyze IgE binding by ELISA, with serum pools of patients from the cities of Datong and Taiyuan in Shanxi province, Beijing, Yantai-Shandong and Qvjing-Yunnan. Further IgE reactivity of each pollen extract of the six Chinese *Artemisia* species was assessed by ELISA, using sera of 142 individual mugwort-allergic patients. ELISA plates (Corning, USA) were coated with 0.5 μg/well pollen extracts in PBS buffer (pH 8.3). After blocking with 100 μl 5% skimmed milk, 100 μl serum pool was added, with a negative serum pool as control. After washing, 100 μl goat anti-human IgE coupled with HRP (1:3000 in PBS buffer) was added and bound IgE was detected using TMB. The ELISA was quantified using the colorimetric reaction at 450/620 nm. We also compared the IgE binding values with rArt v 1.0101 and rArt v 3.0201 allergens tested by ELISA and the values of Art v 1 and Art v 3 sIgE tested by ImmunoCAP. For the patients who were positive in ImmunoCAP with *A. vulgaris* but negative in ELISA with the extracts of six Chinese *Artemisia* spp., IgE binding capacity was further tested using a mixture of pollen extracts of *A. annua*, *A. argyi*, and natural purified Art an 3 and Art ar 3 in a mass ratio of 4:4:1:1 (total of 0.5 μg/well mixture).

Inhibition curves were obtained using inhibitors with serial dilutions of pollen extracts and recombinant *A. vulgaris* allergens in competition with a solid phase coated with rArt v 1.0101 and rArt v 3.0201 for IgE binding, using the serum pools from Shanxi and Shandong. ImmunoCAP inhibition on commercial mugwort (*A. vulgaris*) extract was with serial dilutions of pollen extracts from three species (*A. annua, A. sieversiana, A. vulgaris*) against individual serum from four groups of different sensitization patterns (Art v 1 and Art v 3 IgE positive or negative).

### ImmunoCAP tests

According to the sequence diversity, different natural purified allergens from the three groups were selected for testing the IgE by ImmunoCAP. Allergens were biotinylated and coupled to streptavidin‐conjugated ImmunoCAPs (Thermo Fisher Scientific, Uppsala, Sweden) at 37 °C for 30 min and then were tested with sera of 18 individual mugwort-allergic patients.

### Statistical analyses

Data were analyzed by SPSS21.0, with a value of P < 0.05 considered significantly different. Graphs were drawn with GraphPad Prism6.0. The ANOVA model with Tukey’s post hoc test was used to analyze the differences in protein content between seven *Artemisia spp*. Difference in IgE reactivities was analyzed with the Friedman test and Dunn’s multiple comparison test. The Kruskal–Wallis test with Dunn’s test was used to determine the quantitative variables of the three allergen components, and Spearman’s correlation coefficient analysis to evaluate correlations between ImmunoCAP scores and ELISA values. The four-parameter dose–response curve models were used to build the ELISA standard curves.

## Results

### Patients

Among 150 patients (71 male, 79 female; age range 6-62 years old, mean 30.9 ± 14.7 years), 137 (91%) were diagnosed with allergic rhinitis, 43 (29%) with conjunctivitis, 42 (28%) with asthma and 12 (8%) with eczema. For IgE reactivity against the major components, 103 (68.7%) were positive to Art v 1, 74 (49.3%) were positive to Art v 3, and 61 (40.7%) were positive to both, while 24 (16%) were negative to both allergens (Additional file 1: Table S1).

### Sequence variation of Art v 1, Art v 2 and Art v 3 homologous proteins

**Fig. 2:**
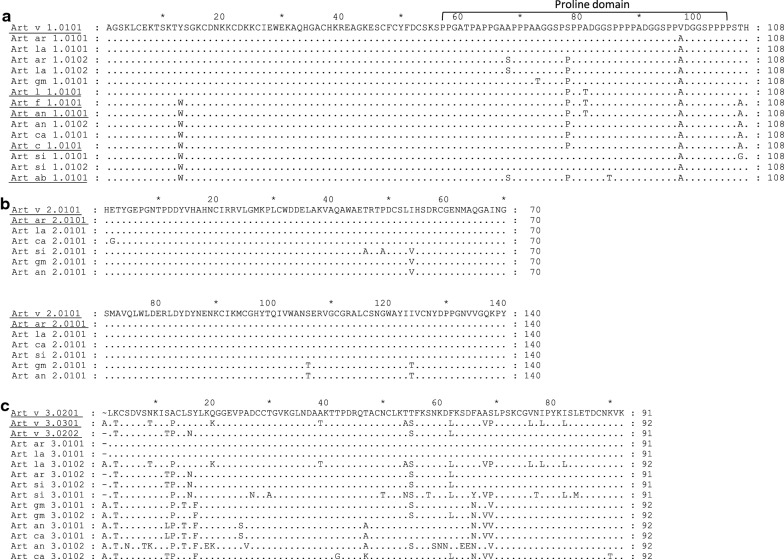
Comparison of pollen allergen deduced amino acid sequences from different *Artemisia* species. **a** defensin-like protein (Art v 1 homologous allergen); **b** pathogenesis-related protein 1 (Art v 2 homologous allergen); **c** non-specific lipid transfer protein (Art v 3 homologous allergen). Underlined sequences indicate the allergens identified in previous research. GenBank accessions are listed in Additional file 1: Table S2

Three types of allergens in seven *Artemisia* spp. were identified by a joint analysis of pollen transcriptome assembly, PCR cloning and sequencing (Additional file 1: Table S2). The natural allergens purified by mAb (Additional file 1: Figure S2) were matched to the target allergen sequences, and no other allergens were found by mass spectrometry (Additional file 1: Figure S3). This gave six new deduced defensin-like (Art v 1 type) proteins in six *Artemisia* spp. in China, with six in the IUIS from reference *A. vulgaris* and the other five species (Fig. 2a). They are highly conserved at the N-terminus, with seven variable amino acids (Fig. 2a). We identified a unique amino acid, 13 W, in the defensin-domain that was present in three of the species from China (*A. annua, A. capillaris, A. sieversiana*) and in four American Artemisia species [29], but not in *A. vulgaris*. In the proline domain, there was 78S/P substitution in *A. vulgaris, A*. *argyi* and *A. lavandulifolia*, while in *A. sieversiana*, only 78S was found, and in *A. annua*, *A. capillaris* and A. *gmelinii* only 78P.

One Art v 2 homologous isoform was obtained from each species, resulting in four different sequences from seven species: Art v 2.0101 (MF326222), Art ar 2.0101 and Art la 2.0101 were identical, as were Art gm 2.0101 and Art an 2.0101, and Art si 2.0101 had an isoform with two extra amino acids (Fig. 2b). The current reference Art v 2.0101 in IUIS was deduced from AM279693, it was not confirmed in this study.

More sequence variations were observed in the lipid transfer proteins (Art v 3 type), with a total of nine isoforms or variants and up to 38 amino acids difference (Fig. 2c). Identical isoforms were found in different species. Two isoforms, Art an 3.0102 and Art si 3.0101, had a few specific amino acids (Fig. 2c). Most isoforms from the six Chinese *Artemisa* spp. were verified by mass spectrometry after immuno-affinity purification of targeted allergens. Art v 1.0101, Art v 2.0101, Art v 3.0201, Art v 3.0202 and Art v 3.0301 were confirmed in the reference *A. vulgaris*, while Art v 3.0101 partial sequences were not. Rather it appeared in Art si 3.0101, because a unique peptide QGGEVPADCCAGVK was found.

### Quantification of pollen extracts and three components

**Fig. 3:**
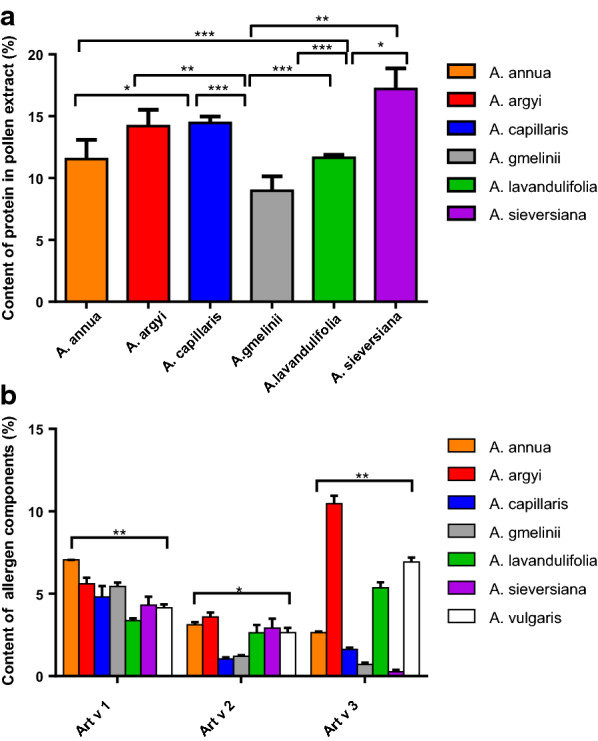
Content of protein in pollen extracts (**a**) and three allergen components in extracts from seven *Artemisia* spp. **b** Differences between groups were analyzed by Tukey post hoc test (**a**) and Kruskal–Wallis test with Dunn’s test (**b**) *P < 0.05, **P < 0.01, ***P < 0.001

Total extracted protein per gram pollen weight from the seven *Artemisa* spp. ranged from the lowest 90 mg in *A. gmelinii* to the highest, 172 mg, in *A. sieversiana* (Fig. 3a). Standard ELISA quantification curves were established for different allergens and isoforms (Additional file 1: Figure S4), giving a range of homologous allergen content of single allergen components in protein extracts from seven species: Art v 1 ranged from 3.4% in *A. lavandulifolia* to 7.1% in *A. annua*; Art v 2 from 1.0% in *A. capillaris* to 3.6% in *A. lavandulifolia*, and Art v 3 from 0.3% in *A. sieversiana* to 10.5% in *A. argyi* (Fig. 3b). The yield of natural allergens purified by mAb was approximate in accordance with the result obtained by ELISA quantification (Additional file 1: Table S3), while the productivity was significantly lower than expected because of a certain amount of loss during the purification for highest purity.

### IgE binding comparison

**Fig. 4:**
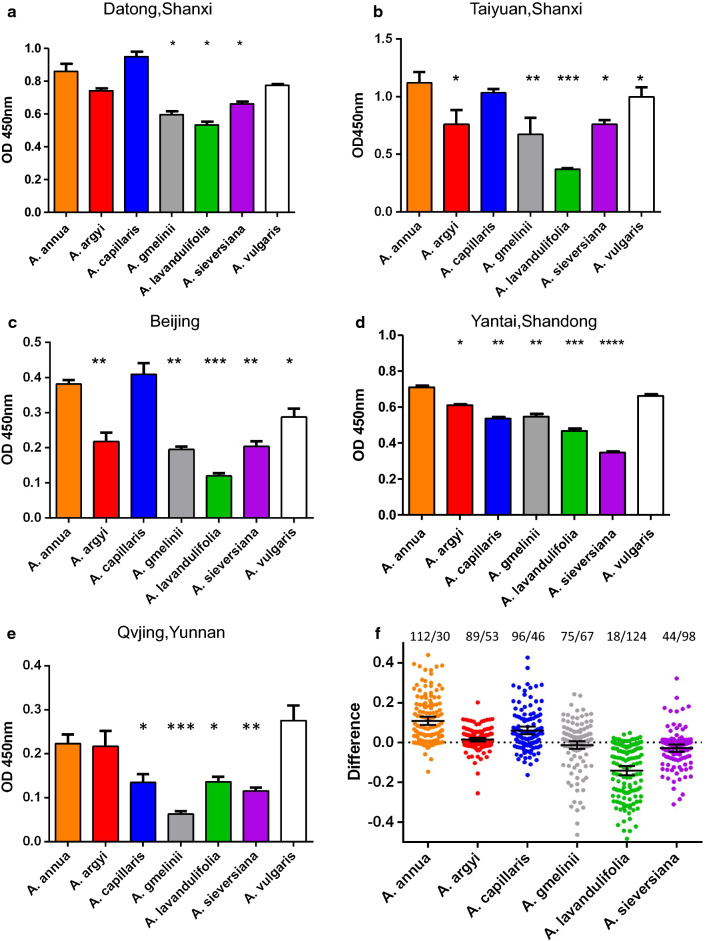
IgE-reactivity of *Artemisia* pollen extracts with serum pools from Datong-Shanxi (**a**), Taiyuan-Shanxi (**b**), Beijing (**c**), Yantai-Shandong (**d**) and Yunnan (**e**) as determined by ELISA.ImmunoCAP IgE characterization of serum pools is shown in Additional file 1: Table S4. **f** IgE-reactivity of 142 individual sera to six *Artemisia* spp is shown as the OD deviation from the average of the six Artemisia spp. Black lines indicate mean OD difference. x/y numbers indicate the number of sera with higher (x) or lower OD (y) than the average of the six species

Using the serum pools from the five areas in China, we compared the IgE binding of six Chinese *Artemisia* spp. with the reference extract *A. vulgaris.* We demonstrated that the IgE binding capacity of *A. annua* and *A. vulgaris* was significantly higher than that of *A. gmelinii*, *A. lavandulifolia* and *A. sieversiana*. The IgE binding potency of *A. capillaris* varied in the five areas: highest in Datong-Shanxi and Beijing (Fig. 4a, c), and significantly lower in Shandong and Yunnan compared to *A. annua, A. argyi* and *A. vulgaris* (Fig. 4d, e). The IgE binding of 142 individual sera to pollen extracts from six Chinese mugwort species again demonstrated higher IgE reactivity to *A. annua* than to the other *Artemisia* spp., with *A. lavandulifolia* and *A. sieversiana* the lowest (Fig. 4f).

**Fig. 5:**
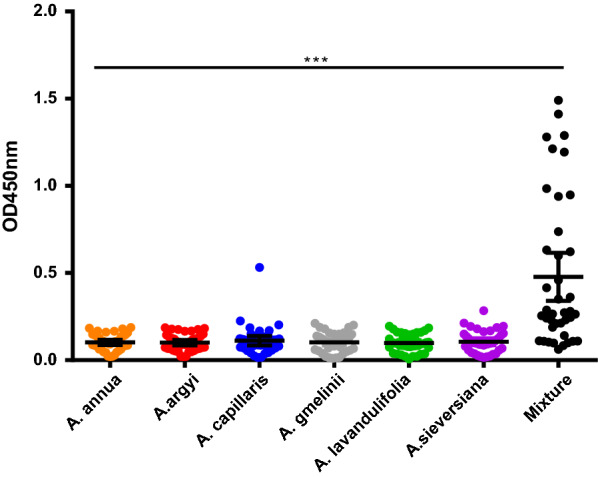
IgE reactivity to six Chinese *Artemisia* pollen extracts and a mixture containing extracts and mAb purified nArt an 3 and nArt ar 3 from serum of the 41 patients

Of 142 mugwort allergic patients, 39 showed negative IgE reactivity to six Chinese mugwort pollen extracts in ELISA, and in one patient only *A. capillaris* was recognized and in another only *A. sieversiana*. These 41 patients had significantly lower IgE reactivity to mugwort extract and Art v 1 and a slightly higher IgE reactivity to Art v 3 in ImmunoCAP. After testing with the mixture of extracts spiked Art an 3 and Art ar 3, in 30 of these 41 patients there was positive IgE binding, especially with the Art v 3 positive patients (IgE reactivity to the mixture was positive in 17/19). The response in the 11 remaining patients was still negative to the mixture (Fig. 5), these patients were negative to Art v 1, and the IgE reactivities to mugwort extract (w6 range: 0.46-5.8) and Art v 3 (w233 range: 0-2.7) was low.

**Fig. 6:**
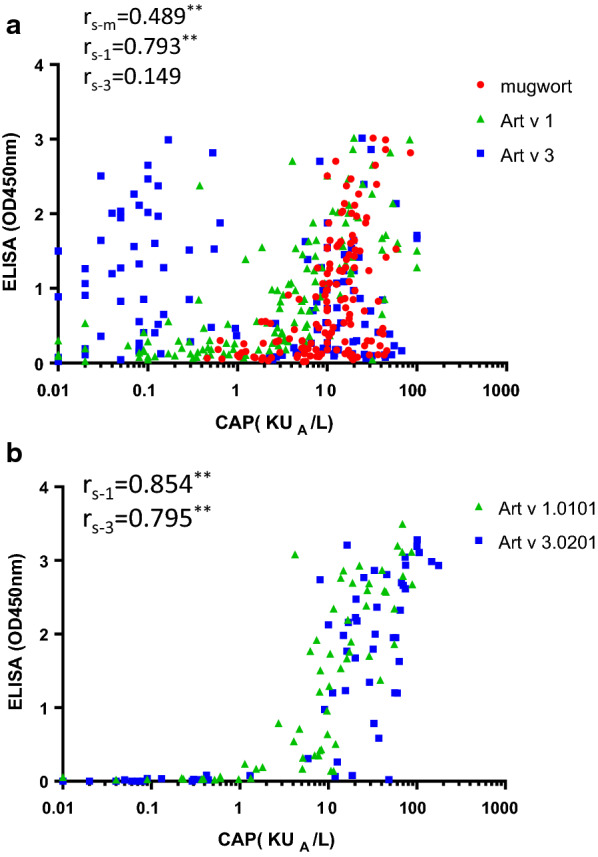
Correlation between ImmunoCAP scores and ELISA values. **a** mugwort CAP (rs-m), Art v 1 CAP (rs-1), Art v 3 CAP (rs-3) scores with averaged ELISA scores from six Artemisia pollen extracts; **b** Art v 1 CAP (rs-1), Art v 3 CAP (rs-3) scores with ELISA coated with recombinant Art v 1.0101 and Art v 3.0201

The IgE binding strength to mugwort extract of the 142 individual patients, measured as ELISA OD values, was closely related to the nArt v 1 IgE ImmunoCAP score, but not to the nArt v 3 score (Fig. 6a). However, when rArt v 1.0101 and rArt v 3.0201 were coated in the ELISA assay, there was good correlation for both components (Fig. 6b).

**Fig. 7:**
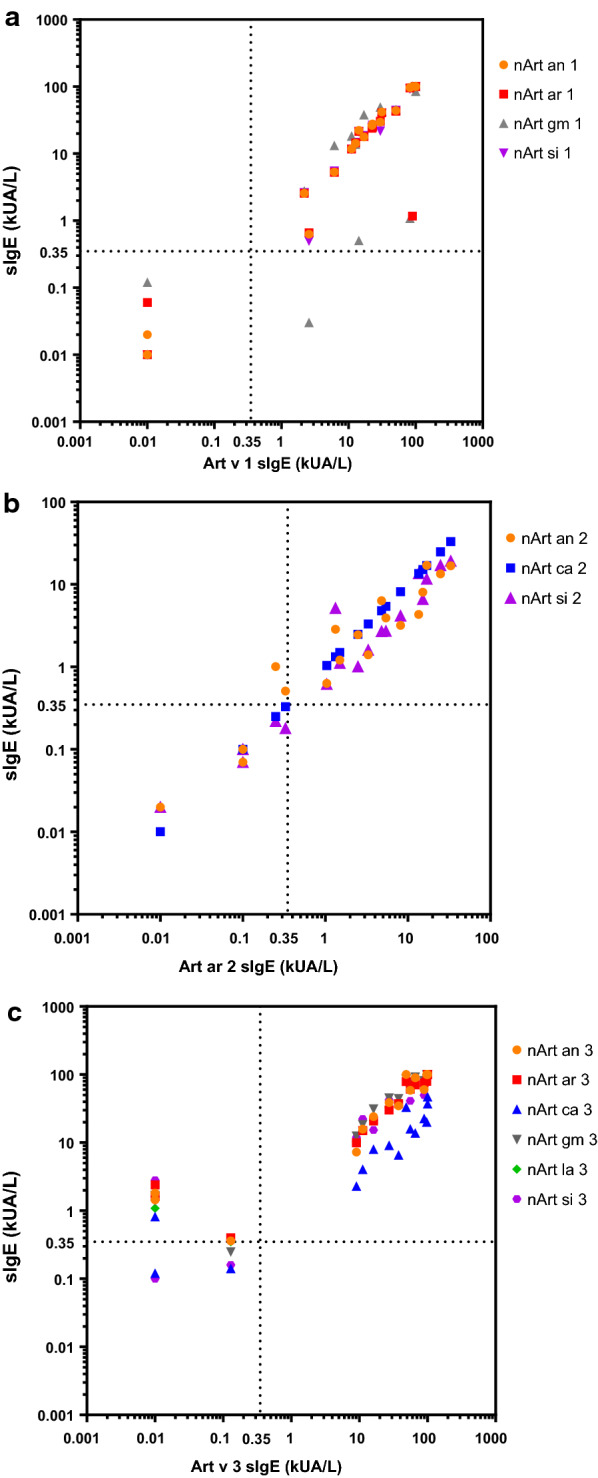
IgE levels to natural Art v 1 (**a**), Art ar 2 (**b**) and Art v 3 (**c**) homologous allergens of the same mugwort-sensitized patients in China tested by ImmunoCAP. The cutoff value (0.35 kUA/L) was indicated by dashed lines

By testing IgE reactivity of natural Art v 1, Art v 2 and Art v 3 homologous allergens by the ImmunoCAP system, we found that the IgE positive rates and values were quite similar for these allergens with high sequence identity, such as Art v 1 homologues (Fig. 7a), for Art v 2 type, Art ar 2 and Art ca 2 were slightly higher than Art si 2 and Art an 2 (Fig. 7b), while Art v 3 homologues were more variable, Art ca 3 was significantly lower, and the positive rates and IgE values of Art an 3, Art ar 3, Art gm 3, Art la 3 and Art si 3 were higher than Art v 3 and Art ca 3 (Fig. 7c).

### IgE inhibition using ImmunoCAP

**Fig. 8:**
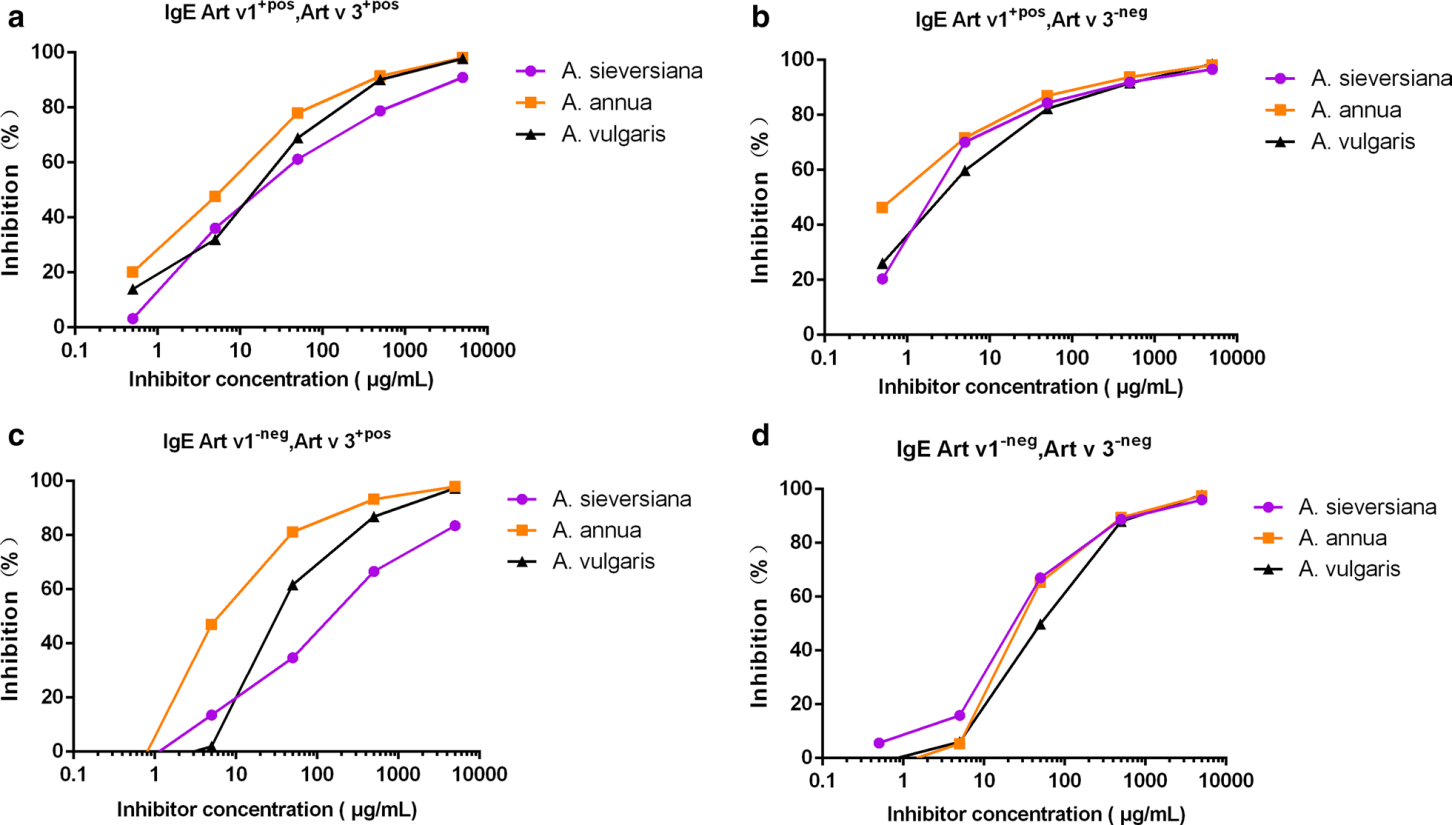
ImmunoCAP inhibition of *A. annua*, *A. sieversiana* and *A. vulgaris* against *A. vulgaris* pollen extract with four groups of Artemisia allergic patients. **a** patients sensitized to both Art v 1 and Art v 3; **b** patients sensitized to Art v 1 but not Art v 3; **c** patients sensitized to Art v 3 but not Art v 1; **d** patients not sensitized to either Art v 1 or Art v 3.X-axis is the inhibitor concentration of pollen extracts

Mugwort ImmunoCAP assays with *A. annua*, *A. vulgaris* and *A. sieversiana* extracts on 16 different patients of the four groups (sensitized to Art v 1 and Art v 3 positive or negative) confirmed that in the Art v 3 positive sera group, the IgE inhibiting capacity was higher with the *A. annua* extract and lower with *A. sieversiana*, especially when Art v 1 was negative (Fig. 8a, c), but this was not the case in the Art v 3 negative sera group (Fig. 8b, d). In patient DT22 (component profile of high Art an 7 IgE and positive Art ar 2), IgE inhibition was even higher with *A. sieversiana*. These results indicate that the IgE binding potency was dependent on the presence of specific allergen molecules in the extract. Using ELISA to test for inhibition to rArt v 1.0101 and rArt v 3.0201 with seven pollen extracts, using serum pools from Shanxi and Shandong, again a large difference was found. Inhibition to both allergen molecules and in two areas was highest with the *A. argyi* extract and lowest with *A. sieversiana* compared to the other species (Additional file 1: Figure S5). In general, there was cross-reactivity in ELISA assays coated with different *Artemisia* spp. extracts, except for *A. sieversiana* (Additional file 1: Figure S6). This suggests that *A. sieversiana* pollen is not the primary sensitizing source.

## Discussion

Here we present a comprehensive analysis of three group allergens, with amino acid sequence, quantity measurement and IgE binding strength of pollen extracts, from seven *Artemisia* spp. These species are representative of four sections of botanical classification and distribution in China.

The degree of allergen sequence variation in different *Artemisa* spp. is related to the phylogenic classification, being similar if they belong to the same section, such as *A. vulgaris, A. argyi*, and *A. lavandulifolia* (Fig. 2). Both Art v 1 and Art v 2 homologous allergen sequences in the seven *Artemisia* spp. were highly conserved, with only a few amino acid changes, indicating a general cross reactivity in all species of this genus (Fig. 2a, b). Art v 3 type is more variable: 26 new amino acid differences were found, mainly in *A. annua* and *A. sieversiana*. Including Art an 7 type sequences investigated in a previous study [17], the amino acid sequences of four allergens in the seven *Artemisa* spp. indicated phylogenic relationship and fit into four botanically classified sections of this genus: Artemisia, Abrotanum, Dracunculus and Absinthium. A recent report on the Art v 1 group allergen sequences from American mugwort (*A. ludoviciana A. californica, A. frigida,* and *A. tridentate* belong to Tridenta section) showed additional amino acid variations, 81T and 85T, in the proline domain (Fig. 2a) [29].

Previous sequencing of cDNAs from *A. vulgaris* (pollen source assumed to be a single species) identified seven Art v 1 isoforms and four Art v 3 [21, 23], while we deduced one or two variants/isoforms from each species by gene cloning and transcript assembly, verified by proteomic mass spectrometry to isoform level. Mass spectrometry of natural Art v 1 from *A. vulgaris* purified by mAb could be matched to Art v 1.0101, while the natural allergen Art v 3 in *A. vulgaris* purified by mAb could be matched to isoallergens Art v 3.0201 and Art v 3.0301, and the first partial Art v 3.0101 peptide (37aa) to *A. sieversiana*. Since all except *A. lavandulifolia* are diploid [30], there are putatively two variants for each species. We suspect the pollen sources used in previous research were not from a single species, *A. vulgaris*, but mixed with *A. sieversiana*, commonly distributed in Europe. In this study, comprehensive transcript analysis, gene specific cloning and identification of the allergen protein by mass spectrometry guaranteed reliability.

The first evaluation of in vitro cross-reactivity, among nine *Artemisia* spp., was done in the USA [1]. This showed the inhibitory capacity of two local *Artemisia* spp. (*A. biennis*, *A. tridentate*) was greater than that of *A. annua* and *A. vulgaris*, and *A. ludoviciana* the least potent. There was no difference in IgE binding capacity between *E. coli*-expressed recombinant Art v 1 isoforms or Art v 3 isoforms within *A. vulgaris* because the sequences were identical [21, 23]. From our results on sequence diversity, we expect little difference in the Art v 1 homologous isoforms, possibly with greater differences in Art v 2 and Art v 3 homologous isoforms in species such as *A. annua* and *A*. *sieversiana*. When rArt v 1.0101 and rArt v 3.0201 were used as coating antigens, ELISA inhibitions with different *Artemisia* species were not in agreement with the results of Art v 1 and Art v 3 homologues quantification, but related to the sequence similarity of the coated isoform (Additional file 1: Figure S5 and Fig. 2), indicating the potential impact of isoforms on IgE binding.

IgE binding strength of the pollen extract is largely dependent on the quantity of major allergens in the extract and the sensitization profile of a patient’s serum to a single component. The concentrations of pollen extract influence the sensitivity and specificity of diagnosis [31]. Here we found that IgE reactivity of six Chinese *Artemisia* spp. measured by ELISA was mainly related to the Art v 1 homologues content in extracts: *A. argyi* and *A. lavandulifolia* pollen have almost identical sequences in four groups of allergens, but the content is different in Art v 1, causing significantly lower IgE binding of *A. lavandulifolia*. Natural pollen extract is not sufficient to measure all component IgEs, especially for Art v 3 type (Figs. 6a and 8c) where there is low content, and there are other interfering factors, such as IgG antibodies [32]. Moreover, for the 41 mugwort allergic patients who gave negative IgE reactivities to Chinese mugwort pollen extracts by ELISA, 30 gave positive results when coated with a mixture containing extracts spiked with mAb purified nArt an 3 and nArt ar 3 (Fig. 5**)**. This indicated again that the pollen extracts alone were not suitable for in vitro IgE diagnosis, because of the low content of some major and minor allergen molecules in pollen extracts, in addition, for the patients with sensitization to minor allergens alone, using extracts for immunotherapy may not succeed or even worse [33].

The commercial diagnostic from European mugwort *A. vulgaris* was quite similar to Chinese silver mugwort, *A. argyi*, in allergen sequence and in IgE binding potency. Two Chinese mugwort species are worthy of attention: *A. annua* and *A. sieversiana*, both with more sequence variability than the reference *A. vulgaris*. The IgE binding capacity of *A. annua* was also equivalent or slightly higher than that of *A. vulgaris*, while that of *A. sieversiana* was significantly lower. We consider that the IgE binding capacity is determined by the quantity of the major allergens, especially Art v 1, in given pollen extract. Sequence variations in the critical locations are very important, as illustrated in the Amb a 1 isoforms with distinct immunological features [34]. In our research, the IgE values were almost the same in the allergens with high sequence identity (Fig. 7), while for Art v 3 type, the positive rates and IgE reactivity in the five Chinese species except *A. capillaris* were higher than *A. vulgaris*: it is probable that *A. vulgaris* was not the primary sensitizer for Chinese patients. Recombinant isoallergens with large amino acid variations from different species need to be evaluated in a large number of representative sera from different geographic areas to get a more comprehensive view. In different geographic regions, there are different dominant *Artemisia* species with varying flowering time. Pollen peaks and Art v 1 content levels have been reported as higher during *A. campestris* flowering than that of *A. vulgaris* [35]. Choosing the most relevant species in specific areas could improve the accuracy and efficiency of diagnosis. The three allergen quantification methods established in this study could be applied in monitoring the Artemisia pollen allergen exposure and association analysis to allergy symptoms.

## Conclusions

The commercial European mugwort ImmunoCAP (*A. vulgaris*) extract has entered the Chinese diagnostics market, and this research indicates its general suitability in China as in vitro test. Our study demonstrated that *A. sieversiana*, the current laboratory-based mugwort pollen extract used for diagnosis in China, is not sufficient due to the low concentration of major allergen Art v 3 type in extract, especially for those patients who are sensitized to Art v 3 homologous allergens. *A. annua* and *A. argyi* pollens are potentially suitable sources for both diagnosis and immunotherapy, the former extract has been chosen as a sublingual immunotherapy product for seasonal allergic rhinitis [36]. There is high sequence identity of the major mugwort allergens in seven different mugwort species which are common in China. Differences in IgE binding capacities among pollen extracts from the seven mugwort species were mainly due to variations in the quantity of major allergens. We therefore consider that purified mugwort pollen allergen components from *A. annua* and *A. argyi* are better suited for diagnosis and treatment than crude pollen extracts which have considerable variations in IgE binding capacity and major allergen content.

## Supplementary information

**Additional file 1: Table S1.** Clinical and demographic data of 150 mugwort pollen-allergic individuals sIgE against mugwort extract (w6), Art v 1 (w231) and Art v 3(w233) determined by ImmunoCAP, ND, not determined. *AS* asthma; *AR* allergic rhinitis; C, conjunctivitis; E, eczema. I-1, I-2, I-3, I-4 indicates the patients serum used in ImmunoCAP inhibition assay belonging to four groups of different sensitization patterns (1, Art v 1 and Art v 3 positive; 2, Art v 1 positive, Art v 3 negative; 3, Art v 1 negative, Art v 3 positive; 4, Art v 1 and Art v 3 negative). The 82 patients reported in previously studies^17, 20, 28^ are indicated by an asterisk. **Table S2.** GenBank accession numbers for three allergen groups in seven Artemisia species.**Table S3.** Productivity of the three group allergens purified by specific mAb**. Table S4.** ImmunoCAP IgE characterization of serum pools from five areas**. Figure S1**. Six *Artemisia* species collected from China. **Figure S2.** SDS-PAGE of natural purified Art v 1, Art v 2 and Art v 3 homologous allergens from six Chinese *Artemisia* species. a, natural Art v 1 homologues purified by specific mAb A7-G4-E6; b, natural Art v 2 homologues purified by specific mAb C9-C1 shown in six different gels; c, natural Art v 3 homologues purified by specific mAb A2-B8. **Figure S3.** Mass spectra of natural purified Art v 1(a), Art v 2(b) and Art v 3(c) homologues. The peptides verified by LC–MS/MS are shown in red and highlighted. **Figure S4.** ELISA quantification of three allergen components in *Artemisia* spp. pollen. a, Chinese silver mugwort (*A. argyi*) pollen extract (ArE) in SDS gel and reaction to polyclonal antibodies (pAb) by Western blot; b, ELISA standard curve for Art v 1 allergen (mAb A7-G4-E6 and rabbit pAbs); c, ELISA standard curve for Art v 2 homologous allergen (mAb C9-C1 with rabbit pAbs); d, ELISA standard curve for Art v 3 homologous allergen with two mAbs (mAbs A2-B8 and A9-G10) with representative different isoforms. **Figure S5.**.Inhibition of IgE binding to Art v 1 and Art v 3 with seven pollen extracts using two serum pools. a: inhibition ELISA coated with rArt v 1.0101, serum pool from Datong, Shanxi (nArt v 1: 11 kUA/l); b: inhibition ELISA coated with rArt v 1.0101, serum pool from Yantai, Shandong (nArt v 1: 6.51 kUA/l); c: inhibition ELISA coated with rArt v 3.0201, serum pool from Datong, Shanxi (CAP nArt v 3: 11.27 kUA/l); d: inhibition ELISA coated with rArt v 3.0201, serum pool from Yantai, Shandong (CAP nArt v 3: 9.65 kUA/l). **Figure S6.** Inhibition of sera pool from Shanxi with extract from different species at 100 μg/ml in ELISA coated with 10 μg/ml of different pollen extracts and mixture. 6-mix, mixture of six *Artemisia* spp. extract in the same proportions.

## Data Availability

The new allergens reported in this manuscript, shown in Additional file 1: Table S2, were deposited in GenBank with accession numbers.

## Abbreviation

IUIS: International Union of Immunological Societies
mAb: Monoclonal antibody
pAb: Polyclonal antibody
ELISA: Enzyme-linked immunosorbent assay
