# Supplementary material for: Variation in IgE binding potencies of seven Artemisia species depending on content of major allergens
Source: Clin Transl Allergy. 2020 Nov 18;10:50. doi: 10.1186/s13601-020-00354-7 (PMC7677751; doi:10.1186/s13601-020-00354-7)
Supplement: Supplementary file 1 — Additional file 1: Table S1. Clinical and demographic data of 150 mugwort pollen-allergic individuals sIgE against mugwort extract (w6), Art v 1 (w231) and Art v 3(w233) determined by ImmunoCAP, ND, not determined. AS asthma; AR allergic rhinitis; C, conjunctivitis; E, eczema. I-1, I-2, I-3, I-4 indicates the patients serum used in ImmunoCAP inhibition assay belonging to four groups of different sensitization patterns (1, Art v 1 and Art v 3 positive; 2, Art v 1 positive, Art v 3 negative; 3, Art v 1 negative, Art v 3 positive; 4, Art v 1 and Art v 3 negative). The 82 patients reported in previously studies17, 20, 28 are indicated by an asterisk. Table S2. GenBank accession numbers for three allergen groups in seven Artemisia species.Table S3. Productivity of the three group allergens purified by specific mAb. Table S4. ImmunoCAP IgE characterization of serum pools from five areas. Figure S1. Six Artemisia species collected from China. Figure S2. SDS-PAGE of natural purified Art v 1, Art v 2 and Art v 3 homologous allergens from six Chinese Artemisia species. a, natural Art v 1 homologues purified by specific mAb A7-G4-E6; b, natural Art v 2 homologues purified by specific mAb C9-C1 shown in six different gels; c, natural Art v 3 homologues purified by specific mAb A2-B8. Figure S3. Mass spectra of natural purified Art v 1(a), Art v 2(b) and Art v 3(c) homologues. The peptides verified by LC–MS/MS are shown in red and highlighted. Figure S4. ELISA quantification of three allergen components in Artemisia spp. pollen. a, Chinese silver mugwort (A. argyi) pollen extract (ArE) in SDS gel and reaction to polyclonal antibodies (pAb) by Western blot; b, ELISA standard curve for Art v 1 allergen (mAb A7-G4-E6 and rabbit pAbs); c, ELISA standard curve for Art v 2 homologous allergen (mAb C9-C1 with rabbit pAbs); d, ELISA standard curve for Art v 3 homologous allergen with two mAbs (mAbs A2-B8 and A9-G10) with representative different isoforms. Figure S5..Inhibition of IgE bi [file 13601_2020_354_MOESM1_ESM.docx]

**Additional file 1**

**Table S1** Clinical and demographic data of 150 mugwort pollen-allergic individuals

|  |  |  |  |  | **sIgE(kUA/L)** | | | **Experiments**  **performed** |
| --- | --- | --- | --- | --- | --- | --- | --- | --- |
| **Area** | **Patients** | **Gender** | **Age** | **Symptom** | **W6** | **Art v 1** | **Art v 3** |  |
| **Datong-Shanxi** | DT7* | F | 16 | C,AR | 37.2 | 5.23 | 0.05 |  |
|  | DT8* | F | 30 | C,AR | 14.9 | 11.56 | 0.05 | I-2 |
|  | DT9 | F | 34 | C,AR | 3.69 | 3.23 | 0.00 |  |
|  | DT10 | M | 42 | C,AR | 1.18 | 0.93 | 0.00 |  |
|  | DT11* | M | 34 | AR,C,As. | 12.7 | 3.72 | 9.98 |  |
|  | DT12* | F | 22 | AR,C | 20.3 | 17.50 | 0.08 |  |
|  | DT13 | M | 9 | AR,C | 18.8 | 17.30 | 0.04 |  |
|  | DT14 | F | 19 | AR,C | 10 | 5.75 | 10.00 |  |
|  | DT15 | F | 31 | AR,C | 0.64 | 0.49 | 0.00 |  |
|  | DT16* | F | 37 | AR,As | 17.6 | 5.34 | 15.60 |  |
|  | DT17* | F | 12 | AR | 46.2 | 2.27 | 67.80 |  |
|  | DT18 | M | 17 | AR,C | 37 | 0.80 | 52.30 | I-3 |
|  | DT19* | M | 11 | AR,As | 8.14 | 0.12 | 2.28 |  |
|  | DT20* | M | 55 | AR,As | 16 | 8.78 | 8.58 | I-1 |
|  | DT22* | F | 17 | AR,C | 26.2 | 0.06 | 0.00 | I-4 |
|  | DT24* | F | 15 | AR | 20.4 | 4.42 | 19.80 |  |
|  | DT25* | F | 30 | AR,C | 45.3 | 19.30 | 22.60 |  |
|  | DT26* | F | 13 | AR,C | 43.5 | 0.01 | 0.00 | I-4 |
|  | DT27* | F | 40 | AR | 35.6 | 24.80 | 25.70 | I-1 |
|  | DT28* | M | 9 | C | 9.92 | 3.02 | 7.69 |  |
|  | DT30 | F | 30 | AR,As | 2.89 | 3.32 | 0.00 |  |
|  | DT31* | M | 12 | AR | 29.2 | 7.19 | 20.00 |  |
|  | DT34 | M | 20 | AR,C | 4.32 | 0.00 | 7.78 |  |
|  | DT36 | M | 51 | AR | 5.58 | 0.08 | 4.50 |  |
|  | DT37* | F | 25 | AR,C | 27.5 | 22.20 | 0.05 |  |
|  | DT38 | F | 40 | AR,C | 2.06 | 1.64 | 0.00 |  |
|  | DT40 | F | 26 | AR,C | 7.6 | 0.01 | 11.80 |  |
|  | DT41 | M | 16 | AR,C | 2.4 | 0.00 | 0.00 |  |
|  | DT43 | M | 27 | AR,C | 0.46 | 0.00 | 0.00 |  |
|  | DT51 | F | 22 | AR | 1.11 | 0.00 | 0.00 |  |
|  | DT58 | M | 31 | AR | 15.8 | 0.29 | 19.50 |  |
|  | DT59* | M | 55 | AR | 32.5 | 19.90 | 24.50 |  |
|  | DT60* | M | 33 | AR | 20.2 | 7.56 | 19.80 |  |
|  | DT61* | M | 9 | AR,As | 26.8 | 16.10 | 0.64 |  |
|  | DT62* | M | 20 | AR,C | 10 | 4.45 | 18.70 |  |
|  | DT65 | M | 12 | AR | 14.2 | 6.20 | 5.70 |  |
|  | DT66 | F | 52 | AR,As | 5.56 | 0.17 | 0.14 |  |
|  | DT67 | M | 22 | AR | 12.8 | 1.72 | 13.30 |  |
|  | DT68 | M | 20 | AR | 12.9 | 15.00 | 0.07 |  |
|  | DT69 | F | 40 | AR | 7.46 | 8.39 | 0.01 |  |
|  | DT71 | F | 11 | AR | 16.1 | 7.47 | 12.90 |  |
|  | DT72 | F | 24 | AR | 20.2 | 7.37 | ND |  |
|  | DT75 | F | 42 | AR | 14.8 | 18.10 | 0.00 |  |
|  | DT78 | F | 13 | AR | 2.78 | 3.66 | 0.00 |  |
|  | DT79 | M | 50 | AR,As | 11.7 | 5.70 | 0.51 |  |
|  | DT81 | M | 52 | AR,C,As | 10.4 | 10.70 | 0.98 |  |
|  | DT82 | M | 48 | AR | 31.2 | 0.22 | 0.00 |  |
|  | DT84 | F | 45 | AR | 4.62 | 0.08 | 0.01 |  |
|  | DT85 | F | 60 | AR | 0.61 | 0.51 | 0.00 |  |
|  | DT87 | F | 41 | AR | 34.5 | 32.20 | 0.10 |  |
|  | DT89 | M | 31 | AR | 18.3 | 0.78 | 26.40 |  |
|  | DT90 | F | 46 | AR | 4.7 | 1.23 | 3.16 |  |
|  | DT92* | F | 12 | AR | 28.1 | 0.01 | 0.00 |  |
|  | DT93 | M | 35 | AR | 15.8 | 0.70 | 19.20 |  |
|  | DT94 | F | 21 | AR | 9.95 | 5.13 | 6.96 |  |
|  | DT95* | M | 6 | E | 10 | 10.00 | 0.03 |  |
|  | DT96 | F | 45 | AR | 1 | 0.00 | 0.00 |  |
|  | DT97 | F | 34 | AR | 0.67 | 0.00 | 0.00 |  |
|  | DT99 | F | 61 | AR | 8.94 | 9.41 | 0.02 |  |
|  | DT100 | M | 20 | AR | 19 | 0.50 | 26.10 |  |
|  | DT101 | F | 45 | AR | 18.9 | 0.32 | 27.20 |  |
|  | DT102 | M | 18 | AR | 2.33 | 0.02 | 2.65 |  |
|  | DT103** | M | 10 | AS,AR | 10 | 3.45 | 10.00 |  |
|  | DT104* | F | 49 | AR,C | 10 | 1.88 | 10.00 |  |
|  | DT105* | M | 45 | AR | 10 | 2.96 | 10.00 |  |
|  | DT106 | F | 29 | AR | 1.52 | 0.00 | 0.00 |  |
|  | DT107 | F | 28 | AR | 1.91 | 0.00 | 0.00 |  |
|  | DT111 | F | 45 | AR | 8.68 | 0.30 | 12.10 |  |
|  | SZ-3* | M | 48 | AR | 22.4 | 31.70 | 0.07 |  |
|  | SZ-5 | M | 21 | AR | 1.57 | 1.17 | 0.00 |  |
|  | S30 | F | 43 | AR | 26 | 6.35 | 30.50 |  |
|  | S31 | M | 42 | AR | 7.52 | 0.90 | 12.40 |  |
|  | S32* | F | 27 | AR,As | 10.2 | 4.31 | 9.69 |  |
|  | S35 | F | 53 | AR | 7.52 | 0.41 | 13.00 |  |
|  | S36 | F | 21 | AR | 13.4 | 40.90 | 100.00 |  |
|  | S41 | F | 51 | AR,As | 14.4 | 13.20 | 0.10 |  |
|  | S42 | M | 47 | AR | 1.94 | 2.45 | 0.08 |  |
|  | S45* | M | 51 | AR,C | 16.4 | 0.38 | 0.13 |  |
|  | S46* | M | 7 | AR,C | 10.5 | 6.32 | 0.03 |  |
|  | S47 | F | 37 | AR | 15.4 | 2.15 | 14.20 |  |
|  | S48 | F | 40 | AR,As | 5.27 | 5.41 | 21.30 |  |
|  | S49 | M | 16 | AR,As | 16.4 | 1.23 | 6.07 |  |
|  | S51 | F | 29 | AR,As | 12.5 | 4.10 | 8.26 |  |
|  | S53* | F | 26 | AR | 39 | 2.86 | 56.20 |  |
|  | S54 | M | 47 | AR | 17.6 | 7.4 | 15.4 |  |
|  | S57 | M | 40 | AR | 4.88 | 5.56 | 0.09 |  |
|  | S60 | M | 24 | AR,As | 10.6 | 3.98 | 12.80 |  |
|  | S67* | F | 40 | AR | 41.8 | 0.42 | 0.95 |  |
|  | S69 | M | 46 | AR | 5.23 | 0.24 | 9.07 |  |
|  | S70* | F | 22 | AR | 11.2 | 0.09 | 0.09 |  |
|  | S71 | F | 51 | AR,C | 2.97 | 0.36 | 4.73 |  |
|  | S73* | F | 13 | AR,E | 44.7 | 83.50 | 0.17 |  |
|  | S74 | F | 12 | AR,As | 85.2 | 50.46 | 0.53 |  |
|  | S76* | F | 53 | C,AR,As | 10.7 | 16.10 | 0.08 |  |
|  | S77* | M | 8 | AR,C | 15.6 | 53.90 | 58.70 |  |
|  | S78 | M | 21 | AR,As | 6.64 | 0.75 | 6.62 |  |
|  | S79 | M | 49 | AR,As | 10.7 | 5.98 | 0.02 |  |
|  | S80* | M | 46 | G,As | 9.49 | 2.61 | 0.12 |  |
|  | S81* | M | 50 | AR,As | 13.8 | 18.40 | 15.32 |  |
|  | S82* | F | 39 | AR,As | 1.24 | 0.09 | 0.08 |  |
|  | S83 | F | 22 | AR,C,E | 42 | 3.10 | 49.50 |  |
|  | S84* | F | 11 | AR,E | 17.3 | 0.09 | 0.08 |  |
|  | S85 | M | 31 | AR,As | 6.49 | 0.20 | 11.90 |  |
|  | S86 | F | 26 | E | 8.07 | 2.79 | 12.70 |  |
|  | S17 | M | 20 | E,C | 1.04 | 0.10 | 2.70 |  |
|  | S39 | M | 14 | E | 2.68 | 0.10 | 0.30 |  |
|  | DT173 | M | 14 | AR | 43.7 | 44.8 | 0.13 | I-2 |
|  | DT248* | F | 17 | AR | 527 | 0 | 207 | I-3 |
|  | DT140* | F | 52 | AR,C,AS | 73.9 | 0.86 | >100 | I-3 |
|  | DT233* | M | 10 | AR,C,AS | 23.2 | 0.03 | 0.003 | I-4 |
|  | DT220* | F | 33 | AR,C | 51.5 | 0.1 | 0.01 | I-4 |
| **Taiyuan-Shanxi** | TY23* | M | 54 | AS,AR | 10 | 2.42 | 9.90 |  |
|  | TY25* | F | 35 | AS,AR | 29.5 | 0.50 | 44.00 | I-3 |
|  | TY2* | F | 22 | AS | 9.75 | 2.79 | 9.01 |  |
|  | TY3* | F | 26 | AS,E | 21.3 | 21.40 | 0.13 | I-2 |
|  | TY4* | F | 42 | AS,AR | 38.8 | 3.14 | 36.90 |  |
|  | TY7* | F | 42 | AS,AR | 18.4 | 17.30 | 0.10 |  |
|  | TY8 | M | 18 | As | 5.48 | 0.47 | 4.25 |  |
|  | TY9 | F | 18 | AR,C | 1.85 | 0.17 | 0.08 |  |
|  | TY10* | M | 18 | AR,As | 10.4 | 6.96 | 10.10 |  |
|  | TY12 | M | 26 | As | 1.86 | 0.10 | 2.47 |  |
|  | TY14 | M | 26 | As | 44.7 | 19.40 | 30.90 |  |
| **Yantai-Shandong** | Y125 | M | 11 | AR,AS | 58.5 | 61.3 | 0.55 |  |
|  | Y241 | F | 17 | AR,E | 18.35 | 61 | 0.12 |  |
|  | Y242 | F | 53 | AS,C,AR | 10.75 | 14.6 | 0.03 |  |
|  | Y262 | M | 13 | AS | 15.75 | 14.6 | 23.3 |  |
|  | Y304 | M | 8 | AR | 7.55 | 5.97 | 0.13 |  |
|  | YT318 | M | 19 | AS,AR | 6.14 | 0.02 | 0.02 |  |
|  | Y320 | F | 62 | C,AR,E | 9.5 | 14 | 0.02 |  |
|  | Y88 | M | 46 | AR | 255 | 157 | 169 | I-1 |
|  | Y84 | M | 54 | As | 278 | 144 | 145 | I-1 |
|  | Y53 | F | 48 | AR | 91.3 | 104.9 | 0.18 | I-2 |
| **Qvjing-Yunnan** | YN23 | M | 54 | C,AR | 5.44 | 0.003 | 0 |  |
|  | YN25 | F | 35 | C,AR | 2.2 | 0.002 | 0.002 |  |
|  | YN30 | F | 30 | AS,C,AR | 10 | 18.4 | 0.04 |  |
|  | YN32 | M | 11 | C,AR,E | 5.8 | 0.02 | 0.01 |  |
|  | YN39* | M | 44 | C,AR | 21.7 | 0.35 | 44.4 |  |
|  | YN49* | M | 24 | AR | 3.8 | 3.36 | 0.003 |  |
|  | YN52* | F | 41 | C,AR,E | 7.8 | 13.7 | 0.05 |  |
|  | YN55* | M | 10 | C,AR | 1.05 | 0.49 | 0.02 |  |
| **Beijing** | 8293 | M | 29 | AR | 23.2 | >100 | 0.01 |  |
|  | 8722 | F | 28 | AR | 19.17 | 45.2 | >100 |  |
|  | 8741 | F | 31 | AR | 20.13 | >100 | 0.15 |  |
|  | 8819 | M | 21 | AR | 20.5 | 41 | 0.29 |  |
|  | 8985 | F | 46 | AR | 19.45 | 43.6 | 0.29 |  |
|  | 8224 | F | 47 | AR | 17.8 | 41 | 0.15 |  |
|  | 8691 | M | 29 | AR | 17.6 | ND | ND |  |
|  | 8844 | F | 35 | AR | 19.5 | 32 | 0.05 |  |
|  | 8220 | M | 27 | AR | 17.9 | 0.45 | 31.7 |  |
|  | 8739 | F | 38 | AR | 14.2 | 0.37 | 41.4 |  |

sIgE against mugwort extract (w6), Art v 1 (w231) and Art v 3(w233) determined by ImmunoCAP, ND, not determined .

AS, asthma; AR, allergic rhinitis; C, conjunctivitis; E, eczema.

I-1, I-2, I-3, I-4 indicates the patients serum used in ImmunoCAP inhibition assay belonging to four groups of different sensitization patterns (1, Art v 1 and Art v 3 positive; 2, Art v 1 positive while Art v 3 negative; 3, Art v 1 negative while Art v 3 positive; 4, Art v 1 and Art v 3 negative). The 82 patients reported in previously studies^17, 20, 27^are indicated by asterisk.

**Table S2** GenBank accession numbers for three allergen groups in seven Artemisia species.

| **Species** | **Allergen name** | **GenBank**  **nucleotide** | **GenBank**  **Protein** | **UniProt** |
| --- | --- | --- | --- | --- |
| *A. annua* | Art an 1.0101 | KC700033 | AHF71022 |  |
|  | Art an 1.0102 | KR996728 | ANC85006 |  |
|  | Art an 2.0101 | MF326216 | AVD29822 |  |
|  | Art an 3.0101 | KR996738 | ANC85017 |  |
|  | Art an 3.0102 | KR996740 | ANC85018 |  |
| *A. argyi* | Art ar 1.0101 | KR996730 | ANC85008 |  |
|  | Art ar 1.0102 | KR996731 | ANC85009 |  |
|  | Art ar 2.0101 | MF326217 | AVD29823 | A0A2L1DGQ3 |
|  | Art ar 3.0101 | KR996741 | ANC85019 |  |
|  | Art ar 3.0102 | KR996742 | ANC85020 |  |
| *A. capillaris* | Art ca 1.0101 | KR996732 | ANC85010 |  |
|  | Art ca 2.0101 | MF326218 | AVD29824 |  |
|  | Art ca 3.0101 | KR996743 | ANC85021 |  |
|  | Art ca 3.0102 | MN650088 | QIN55516 |  |
| *A. gmelinii* | Art gm 1.0101 | KR996733 | ANC85011 |  |
|  | Art gm 2.0101 | MF326219 | AVD29825 |  |
|  | Art gm 3.0101 | KR996744 | ANC85022 |  |
|  | Art gm 3.0102 | KR996745 | ANC85022 |  |
| *A. lavandulifolia* | Art la 1.0101 | KR996735 | ANC85013 |  |
|  | Art la 1.0102 | KR996736 | ANC85014 |  |
|  | Art la 2.0101 | MF326220 | AVD29826 |  |
|  | Art la 3.0101 | KR996746 | ANC85024 |  |
|  | Art la 3.0102 | KR996747 | ANC85025 |  |
| *A. sieversiana* | Art si 1.0101 | KR996737 | ANC85015 |  |
|  | Art si 1.0102 | KR996738 | ANC85016 |  |
|  | Art si2.0101 | MF326221 | AVD29827 |  |
|  | Art si 3.0101 | KR996748 | ANC85026 |  |
|  | Art si 3.0102 | KR996749 | ANC85027 |  |
| 1. *vulgaris* | Art v 1.0101 | AF493943 | AAO24900 | [Q84ZX5](http://www.uniprot.org/uniprot/Q84ZX5) |
|  | Art v 2.0101 | [AM279693](http://www.ncbi.nlm.nih.gov/nuccore/AM279693) | [CAK50834](http://www.ncbi.nlm.nih.gov/nuccore/CAK50834) | [A6GVD5](http://www.uniprot.org/uniprot/A6GVD5) |
|  | Art v 3.0201 | EU564845 | ACE07186 | C4MGG9 |
|  | Art v 3.0202 | EU564846 | ACE07187 | C4MGH0 |
|  | Art v 3.0301 | EU564847 | ACE07188 | C4MGH1 |

**Table S3** Productivity of the three groups allergens purified by specific mAb

| Productivity（μg /g extract） | *A. annua* | *A. argyi* | *A. capillaris* | *A. sieversiana* | *A. gmelinii* | *A.* *lavandulifolia* |
| --- | --- | --- | --- | --- | --- | --- |
| A7G4E6  (10 mg) | 72.82 | 68.96 | 49.39 | 61.41 | 32.5 | 35.59 |
| C9C1  (6 mg) | 100.78 | 161.08 | 83.87 | 116.32 | 67.31 | 95.58 |
| A2B8  (8.4 mg) | 78.24 | 109.84 | 57.38 | 15.51 | 26.835 | 48 |

**Table S4** ImmunoCAP IgE characterization of serum pools from five areas

| Serum pool | Patients No. | Mugwort (kUA/L) | nArt v 1 (kUA/L) | nArtar 2  (kUA/L) | nArt v 3 (kUA/L) | nArtan 7  (kUA/L) |
| --- | --- | --- | --- | --- | --- | --- |
| Datong, Shanxi | 39 | 8.17 | 3.48 | 0.12 | 5.83 | 0.46 |
| Taiyuan, Shanxi | 50 | 9.00 | 5.17 | 2.10 | 5.75 | 0.29 |
| Beijing | 30 | 6.67 | 4.17 | 1.58 | 2.9 | 0.20 |
| Yantai, Shandong | 38 | 4.66 | 2.34 | 0.81 | 3.66 | 0.26 |
| Qvjing, Yunnan | 27 | 3.15 | 1.31 | 0.15 | 0.40 | 0.59 |

**Figure S1** Six *Artemisia* species collected from China.

**Figure S2** SDS-PAGE of natural purified Art v 1, Art v 2 and Art v 3 homologous allergens from six Chinese *Artemisia* species.

a, natural Art v 1homologues purified by specific mAb A7-G4-E6; b, natural Art v 2 homologues purified by specific mAb C9-C1 shown in six different gels; c, natural Art v 3 homologues purified by specific mAb A2-B8.

**Figure S3** Mass spectra of natural purified Art v 1(a), Art v 2(b) and Art v 3(c) homologues. The peptides verified by LC-MS/MS shown in red and highlighted.

**Figure S4** ELISA quantification of three allergen components in *Artemisia* spp. pollen.

a, Chinese silver mugwort (*A. argyi*) pollen extract (ArE) in SDS gel and reaction to polyclonal antibodies (pAb) by Western blot; b, ELISA standard curve for Art v 1 allergen (mAb A7-G4-E6 and rabbit pAbs); c, ELISA standard curves for Art v 2 homologous allergen (mAb C9-C1 with rabbit pAbs); d, ELISA standard curves for Art v 3 homologous allergen of//with two mAbs (mAbs A2-B8 and A9-G10) with representative different isoforms.

**Figure S5** Inhibition of IgE binding to Art v 1 and Art v 3 with seven pollen extracts using two serum pools.

a: inhibition ELISA coated with rArt v 1.0101, serum pool from Datong, Shanxi (nArt v 1: 11kUA/l);b: inhibition ELISA coated with rArt v 1.0101, serum pool from Yantai, Shandong (nArt v 1: 6.51 kUA/l);c: inhibition ELISA coated with rArt v 3.0201,serum pool from Datong, Shanxi (CAP nArt v 3: 11.27kUA/l); d: inhibition ELISA coated with rArt v 3.0201, serum pool from Yantai, Shandong (CAP nArt v 3:9.65kUA/l).

**Figure S6** Inhibition of sera pool from Shanxi with extracts from different species at 100 μg/ml in ELISA coated with 10 μg/ml of different pollen extracts and mixture. 6-mix, mixture of six *Artemisia* spp. extracts in the same proportions.
